# Immunohistology and remodeling in fatal pediatric and adolescent asthma

**DOI:** 10.1186/s12931-017-0575-0

**Published:** 2017-05-16

**Authors:** Kristiina Malmström, Jouko Lohi, Antti Sajantila, Frode L. Jahnsen, Merja Kajosaari, Seppo Sarna, Mika J. Mäkelä

**Affiliations:** 1 Dept. of Allergy, University of Helsinki and Helsinki University Hospital, PO Box 160, FI-00029 Helsinki, Finland; 2 Dept. of Pathology, University of Helsinki and Helsinki University Hospital, Helsinki, Finland; 3 Dept. of Forensic Medicine, University of Helsinki, Helsinki, Finland; 4 Dept. of Pathology and Centre for Immune Regulation, University Hospital-Rikshospitalet and University of Oslo, Oslo, Norway; 5 Hospital for Children and Adolescents Hospital, University of Helsinki and Helsinki University Hospital, Helsinki, Finland; 6 Dept. of Public Health, University of Helsinki, Helsinki, Finland

**Keywords:** Airway smooth muscle, Eosinophils, Fatal pediatric and adolescent asthma, Histopathology, Reticular basement membrane

## Abstract

**Background:**

Thickening of reticular basement membrane, increased airway smooth muscle mass and eosinophilic inflammation are found in adult fatal asthma. At the present study the histopathology of fatal paediatric and adolescent asthma is evaluated.

**Methods:**

Post-mortem lung autopsies from 12 fatal asthma cases and 8 non-asthmatic control subjects were examined. Thickness of reticular basement membrane (RBM) and percentage of airway smooth muscle (ASM%) mass area were measured and inflammatory cells were counted. Patient records were reviewed for clinical history.

**Results:**

The age range of the cases was from 0.9 to 19.5 years, eight were males and five had received inhaled corticosteroids. Thickened RBM was detected in majority of the cases without any correlation to treatment delay, age at onset of symptoms or diagnosis. In the large airways ASM was clearly increased in one third of the cases whereas the median ASM% did not differ from that in healthy controls (14.0% vs. 14.0%). In small airways no increase of ASM was found, instead mucous plugs were seen in fatal asthma. The number of eosinophils, plasmacytoid dendritic cells, macrophages, and B-cells were significantly increased in fatal asthma cases compared with controls and the two latter correlated with the length of the fatal exacerbation.

**Conclusions:**

The findings highlight the strong presence of eosinophils and mucous plugs even in small airways in children and adolescents with fatal asthma. Thickened RBM was obvious in majority of the patients. Contrary to our hypothesis, increased ASM% was detected in only one third of the patients.

## Background

It is thought that inflammation and remodelling occur together in asthma [1]. Remodelling is characterized by epithelial injury, thickening of reticular basement membrane (RBM), airway smooth muscle (ASM), goblet cell hypertrophy and hyperplasia, and angiogenesis, whereas the inflammation is merely eosinophilic [2].

The thickness of RBM increases naturally during childhood. RBM thickness of cartilaginous bronchi increases rapidly until 6 years of age thereafter slowly until 17 years of age [3]. Thickened RBM was detected in school children with moderate and severe asthma [4–9], in pre-schoolers with severe wheeze and in mild-to-moderate asthma [4–9] but not in children below two years with recurrent lower airway symptoms regardless of lung function [10].

Increased thickness of ASM is seen in severe adult asthmatics especially in large airways [11, 12] and both ASM hyperplasia and hypertrophy contribute [13, 14]. In children ASM hypertrophy and hyperplasia in large airways were described in six children (6–17 years) with severe corticosteroid-dependent asthma [5]. Subsequently ASM hyperplasia and hypertrophy in large airways were present even in moderate-to-severe asthma in children 7–16 years of age [15].

Chronic airway inflammation in asthma is thought to compose of eosinophils, mast cells, and T-lymphocytes. Airway eosinophilia has been contradictory in childhood asthma especially in early disease. However, varying degree of bronchial eosinophilia without increased neutrophils or mast cells was detected in children with severe treatment-resistant asthma [16].

We re-examined autopsied lung sections for remodelling and airway inflammation from Finnish children and adolescents with fatal asthma and compared these to those obtained from healthy age-related controls with accidental death. We hypothesized that RBM and ASM mass are increased in fatal asthma.

## Methods

### Study subjects

Fatal asthma cases were derived from a death certificate study on fatal asthma in children and adolescents 1976–1998 [17]. Lung tissue autopsies were collected from laboratories in Finland. Data on clinical history and treatment was obtained from patient records. Lung autopsies from 8 children with accidental death between 2006–2010, received from medico-legal autopsies, served as controls. Patient records were reviewed for asthma and atopy. A subject was considered to have atopy if atopic eczema, allergic rhino-conjunctivitis or food allergy were reported. Approval for study was obtained from Ministry of Social Affairs and Health, National Supervisory Authority for Welfare and Health, and Ethics Committee for Hospital for Children and Adolescents.

### Tissue preparation

Autopsies were performed using standard medico-legal autopsy protocols. Lung specimen was fixed in formalin before paraffin embedding, microscopic slide preparation and staining. Bronchi, airways with cartilage and hereafter called as large airways, as well as bronchioles, muscularized columnar lined airways without cartilage, less than 0.4 mm diameter and hereafter called as small airways, were analyzed. The *outer luminal* diameter of bronchi was measured from outer layer of bronchial wall outside cartilage whereas the *inner luminal* diameter of bronchi was measured from inner side of epithelial layer. The thickness of bronchial wall was difference of these parameters. Bronchiolar diameter was measured from outer wall of muscular layer.

Due to the retrospective nature of this study, site of the lung samples were no specified and measured indices may have varied in different part of lungs.

### RBM and ASM measurements

Thickness of RBM was measured from Herovici stained sections in two fashions. 1) *Perpendicular method*: representative perpendicular areas were selected for each airway and RBM thickness was manually measured (10–15 individual measurements). 2) *Grid-overlay method*: whole airway circumference was photographed. Measure points were randomly selected using grid-overlay method. Individual measurements varied in number from 50–200.

**Fig. 1:**
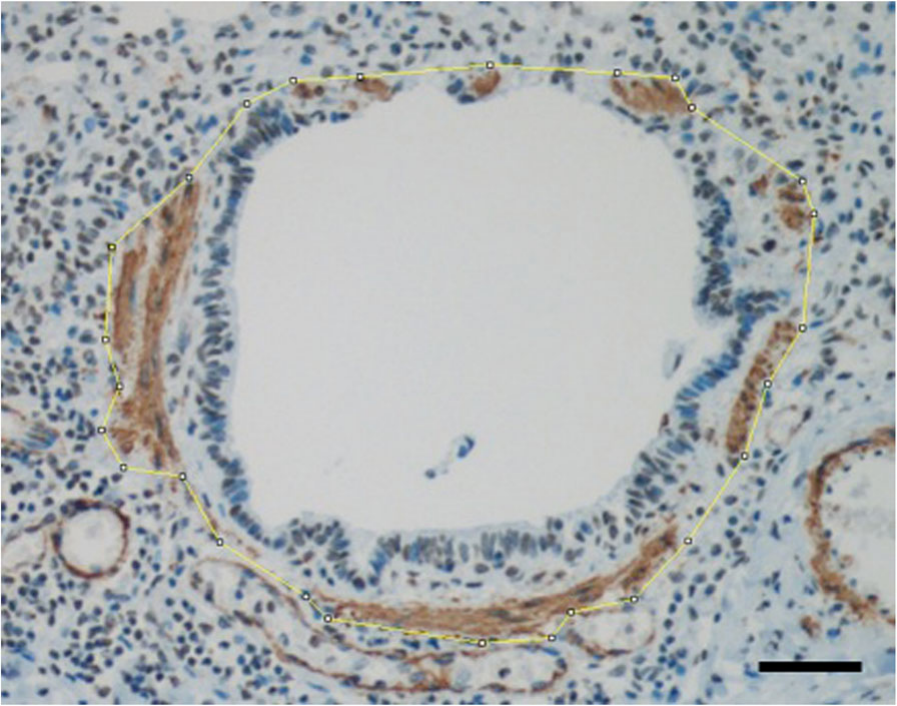
Smooth muscle was quantified from smooth muscle actin stained sections (brown), and expressed as percentage of muscle of total bronchiolar cross sectional area (small airway ASM%), bar 50 um

To measure the amount of ASM samples stained for smooth muscle actin were photographed. The area of airway was determined manually following outlines of smooth muscle layer, Fig. 1. When smooth muscle layer was discontinuous, a straight line was drawn between the nearest visible smooth muscle bundles. If such line intersected the epithelium, the outline was determined by the outline of epithelium. The picture was divided into non-muscle and muscle areas and converted to black and white. To determine if a pixel belonged to the smooth muscle area it was passed through a color threshold filter. Brown-red-colored areas passed as smooth muscle. In some samples epithelium or other cells inside the muscle layer had a red-brown tint and the tinted non-muscle areas were masked with white color before measurement. The amount of smooth muscle is expressed as percentage of cross sectional area of the airway (ASM%). Increased RBM and ASM% were defined as more than one standard deviation above the mean value for control subjects.

### Inflammatory cells and mucus

Inflammatory cells were identified in mucosa and submucosa by immunostaining using antibodies: T-lymphocytes (CD3, 2GVG Ventana, Roche), B-lymphocytes (CD20, L26 Ventana, Roche), plasma cells (CD138, B-A38, Ventana, Roche), mast cells (CD117, polyclonal, Dako), and macrophages (CD163, 10D6, Novocastra). Plasmacytoid dendritic cells (PDC) were identified as CD-123 positive cells (CD123, a mixture of clone 7G3, IgG2a and clone 9 F5, IgG1; BD Pharmingen, CA) with typical plasmacytoid morphology as described [18]. Identification of conventional dendritic cells with anti-CD11c gave variable staining quality and was rejected. Eosinophils were counted from hematoxylin-eosin slides. Neutrophilic leukocytes and eosinophils were stained with CD15 (MMA, Roche) and identified based on morphology. Results were expressed as number of cells/subepithelial area (1/mm^2^). Mucous plug was identified by Alcian Blue-Periodic Acid-Schiff stain and scored semi-quantitatively: 0 = none; 1 = some; 2 = prominent; 3 = obstructive.

### Statistical analysis

Mann-Whitney’s test was used to compare results between the groups and Wilcoxon’s test within the groups for non-normal data. Comparison of means with normally distributed variables was done with *t*-test. The associations between histological and clinical findings were evaluated with Spearman’s correlations and Chi^2^-tests. Two-sided *p*-values <0.05 were considered statistically significant.

## Results

Median age at asthma death was 3.1 years (range 0.9 to 19.5) for 12 cases compared with 5.5 years (range 0.1 to 16.4) for eight controls, Table 1. Of the cases with reliable information, 4/6 cases had parental asthma and 9/9 cases had atopy whereas all controls were non-atopic. Symptoms of acute respiratory airway infection were reported in 9/12 cases. Length of fatal asthma exacerbation, i.e. time from beginning of exacerbation till death, was available in 11/12 cases and the median length was 2.0 days. Inhaled corticosteroids were used regularly by 5/12. Median delay of any anti-inflammatory asthma medication from beginning of symptoms was 0.8 years.

**Table 1:** Demographics of the fatal asthma cases

Sex	Age at death (y)	Age at onset of symptoms (y)	Lifetime duration of symptoms (y)	Age at diagnose (y)	Delay of any anti-inflammatory asthma medication (y)	Length of treatment (y)	ICS	Other regular asthma therapy	Atopy
F	1,9	1,1	0,8	NA	0,8	0,0	–	–	NA
M	18,9	0,5	18,4	3	6,5	11,9	–	T, C	1
M	0,9	NA	NA	NA	NA	NA	NA	NA	NA
M	1,9	1,1	0,8	1,5	0,4*	0,4*	–	–	1
F	2,6	0,7	1,9	0,8	0,1	1,8	1	T, C	1
M	18,1	1,0	17,1	1,3	12,0	5,1	–	T	1
M	19,5	1,0	18,5	3	3,0	15,5	1	T, C	1
F	6,0	2,2	3,8	NA	3,8	0,0	–	–	1
M	2,4	0,7	1,7	0,9	0,2	1,5	1	T	1
F	1,8	1,5	0,3	1,7	0,2	0,1	–	T	1
M	18,7	15,0	3,7	17,8	2,8	0,9	1	LABA	1
M	3,6	0,8	2,8	0,9	0,1	2,7	1	C	1
Median	3,1	1,0	2,8	1,5	0,8	1,7			

*Abbreviations: ICS* inhaled corticosteroids, *T* theophyllin, *C* cromoclygate, *LABA* long-acting beta-agonist, *NA* non-available information

*Theophyllin was given at exacerbations during the last five months

The number of identified large and small airways was 1–4 and 2–15 per sample. Median diameters of these airways are presented in Table 2. Individual remodelling and immunohistological findings are presented in Table 3.

**Table 2:** Remodelling and immunohistological findings

	Fatal asthma cases	Healthy controls	p*
RBM, um [median, (IQR)]^a^	5,7 (2,8)	2,3 (1,3)	**0.001**
RBM, um [median, (IQR)]^b^	5,3 (1,8)	3,4 (0,8)	**0.002**
ASM large AW, %, [median, (IQR)]	15,1 (15,6)	15 (3,5)	0.933
ASM small AW, % [median, (IQR)]	14,0 (7)	14,0 (8)	0.553
T cells [median, (IQR)]	197 (159)	213/126)	0.866
B cells [median, (IQR)]	43 (95)	19,3 (18)	**0.028**
Macrophages [median, (IQR)]	216 (110)	93 (22)	**0.001**
Mast cells [median, (IQR)]	73 (23)	75 (220)	0.8
CD15 + cells [median, (IQR)]	53 (187)	65 (130)	0.671
Eosinophils [median, (IQR)]	120 (220)	0 (4)	**<0.001**
Plasmacytoid dendritic cells, [median, (IQR)]	20 (30)	4 (10)	**0.012**
Mucus bronchi [median, (IQR)]	2 (1)	1 (1)	**<0.001**
Mucus bronchioles [median, (IQR)]	1 (0)	0 (0)	**<0.001**
Outer luminal diameter, large AW, mm [median, (IQR)]	2,0 (1,1)	1,5 (0,8)	0.069
Inner luminal diameter, large AW, mm [median, (IQR)]	1,0 (0,4)	0,9 (0,4)	0.353
Wall thickness, large AW, mm [median, (IQR)]	0,9 (0,9)	0,7 (0,4)	0.103
Luminal diameter, small AW, mm [median, (IQR)]	0,3 (0,1)	0,2 (0,1)	0.472

*Abbrevations: RBM* reticular basement membrane, *IQR* interquartile range, *ASM* airway smooth muscle, *AW* airways

*Mann Whitney’s test for continuous variables

All the bolded *p*-values are of significance (they are < 0.05)

^a^Perpendicular method

^b^Grid overlay method

**Table 3:** Individual remodelling and immunohistological findings

RBM, um^b^	RBM, um^c^	ASM% large AW	ASM% small AW	T-cell^a^	B- cell	MF	Mast	Eos	CD15	PDC	Mucus large AW (0–3)	Mucus small AW (0–3)
Cases												
2,8	4,4	13	11	121	118	431	129	27	23	0	2,5	1
5,6	5,6	29	17	309	30	345	353	65	140	4	2	1
2,3	3,4	7	9	1097	560	875	154	1240	659	100	3	1
4,0	3,9	9	17	297	64	342	149	77	80	4	2	1
6,9	6,3	15	20	191	49	455	92	240	140	20	3	2
5,8	6,2	20	14	393	87	300	113	653	73	80	2	1
6,4	5,7	28	14	521	19	239	106	360	87	20	3	0
3,4	4,6	12	8	508	225	500	149	20	813	24	2	1
6,4	4,7	14	18	421	443	1133	153	320	107	20	3	1
4,8	5	10	NA	166	51	320	151	700	140	14	3	1
6,6	6,4	17	13	150	35	161	79	1	9	20	3	2,5
6,2	5,6	35	19	427	194	520	146	467	447	40	2	1
Controls												
2,3	3,8	6	6	NA	22	158	140	0	640	0	0	0
3,1	4,9	16	14	454	29	184	248	0	147	0	1	0
1,7	3,3	16	22	257	25	219	169	0	73	12	0	0
3,2	3,6	18	17	487	51	204	95	6	47	4	1	0
1,7	2,6	14	9	400	81	167	131	12	320	8	1	0
2,8	3,4	14	11	450	48	188	187	0	347	4	0	0
1,8	2,4	11	14	68	12	157	65	0	113	12	1	0
2,4	3,3	NA	NA	325	NA	144	NA	0	120	4	0	0

*Abbreviations: RBM* reticular basement membrane, *ASM* airway smooth muscle, *AW* airways, *MF* macrophage, *PDC* plasmacytoid dendritic cell, *NA* non-available information

^a^All cell counts are per mm^2^

^b^Perpendicular method

^c^Grid overlay method

### Remodelling

**Fig. 2:**
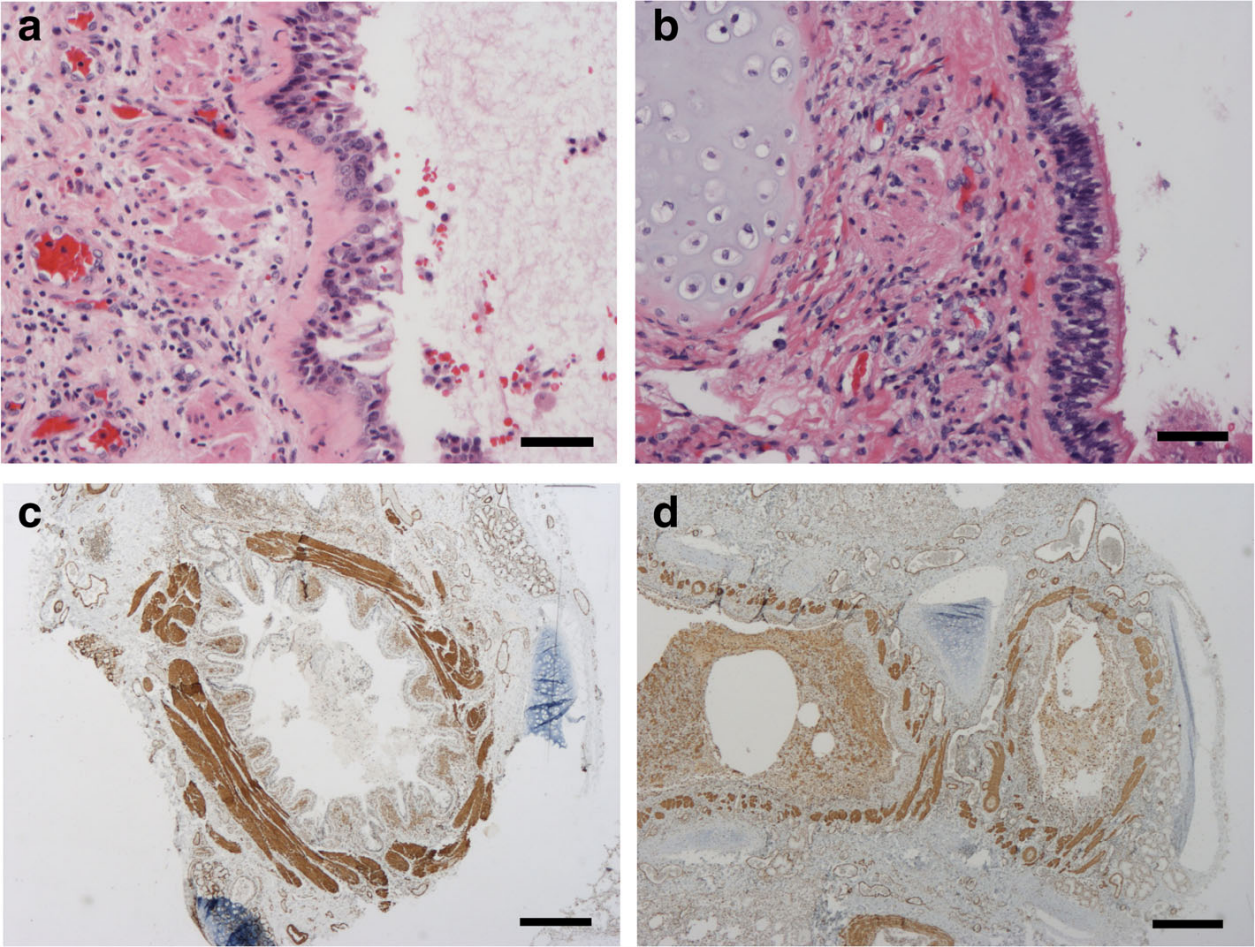
**a** Bronchial thickness of reticular basement membrane (RBM) (red) is increased in a 2.6 year old fatal asthma case (mean 6.9 um) whereas (**b**) the thickness of RBM is normal in a 2.5 year old control (mean 1.7 um) (hematoxylin-eosin stain; bars 50 um). **c** Increased ASM (brown) in a large airway in 19.5 year old fatal asthma case (mean ASM% 28%) compared with (**d**) that in a 6.0 year old fatal asthma (mean ASM% 12%) (smooth muscle actin stain; bars 250 um)

**Fig. 3:**
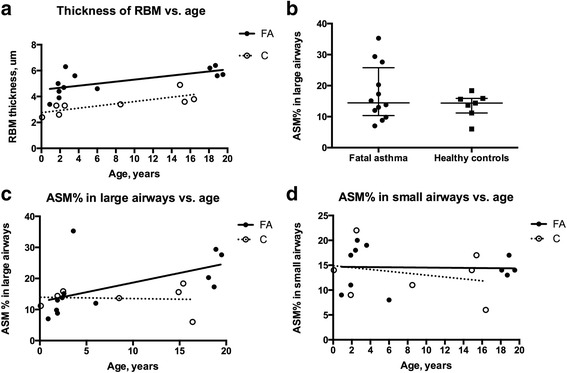
**a** Thickness of RBM increased significantly with age both in fatal asthma (FA) 0.079 um/year (*r* = 0.698; *p* = 0.014, Spearman) and in controls (C) 0.085 um/year (*r* = 0886; *p* = 0.006 Spearman). RBM measured by grid-overlay method. **b** Distribution of ASM% in large airways in fatal asthma (FA) and in healthy controls (C). **c** ASM% in large airways increased significantly with age (0.6%/year) in fatal asthma (FA) (*r* = 0.787; *p* = 0.003, Spearman) but not in controls (C) (−0.04%/year) (*r* = 0.145; *p* = 0.762, Spearman). **d** ASM% in small airways did not change over time in fatal asthma (FA) (0.1%/year) nor in controls (C) (−0.2%/year)

Thickness of RBM in large airways was significantly increased in fatal asthma cases compared with controls, by both perpendicular and grid overlay methods (*p* = 0.001 and *p* = 0.002, Mann Whitney), Table 2, Figs. 2a&b. Thickness of RBM increased significantly with age in both groups, Fig. 3a. Thickness of RBM in fatal asthma did not correlate with any other clinical parameter presented in Table 1.

ASM was clearly increased in large airways in 4/12 fatal asthma cases, Figs. 2c&d, but median ASM% did not differ from that in controls (15.1% vs. 15.0%) Fig. 3b. The ASM% in large airways increased with age (*r* = 0.802; *p* = 0.003, Spearman) Fig. 3c and correlated with RBM in fatal asthma (RBM by grid-overlay method *r* = 0.718; *p* = 0.011 and by perpendicular method *r* = 0.601; *p* = 0.039, Spearman). ASM% in large airways correlated significantly with lifetime duration of asthma symptoms (*r* = 0.715; *p* = 0.017, Spearman).

**Fig. 4:**
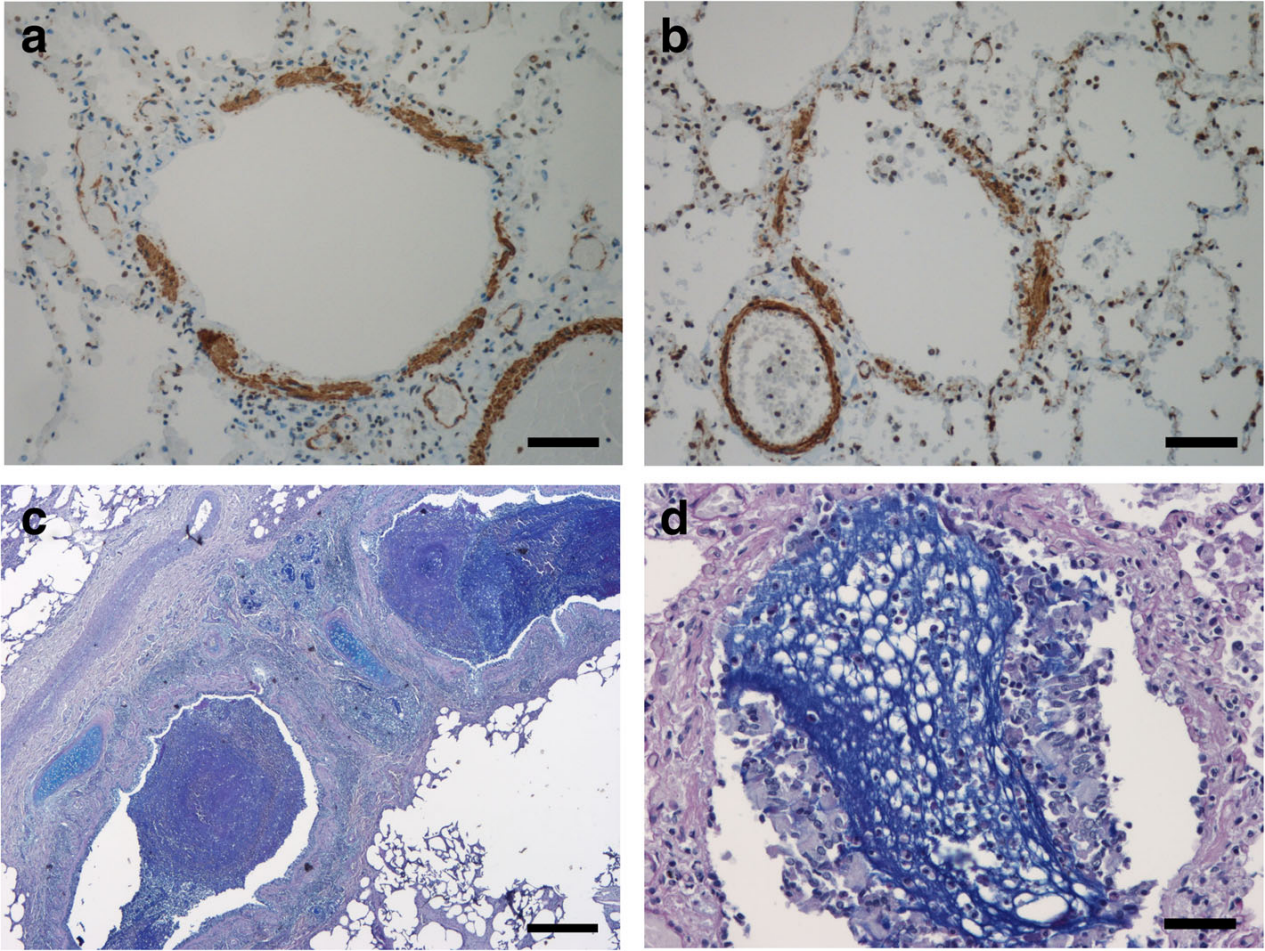
**a** Thickness of ASM in a small airway (brown), in a 19.5 year old fatal asthma case (mean ASM% 14%) is similar to (**b**) that in a 14.9 year old control (mean ASM% 14%) (smooth muscle actin; bars 50 um). **c** Large airway (bronchial) lumen in a 2.4 year old fatal asthma case filled with mucous (Alcian Blue-Periodic Acid-Schiff; bar 250 um). **d** Small airway (bronchiolar) lumen filled with mucous (blue) in a 18.7 year old fatal asthma case (Alcian Blue-Periodic Acid-Schiff; bar 50 um)

ASM% in small airways was found equally in both groups (median 14.0% vs. 14.0%), Figs. 4a&b without any increase with age Figs. 3d. ASM% in small airways correlated negatively with age at the onset of asthma symptoms (*r* = -0.794; *p* = 0.004, Spearman).

### Inflammation

**Fig. 5:**
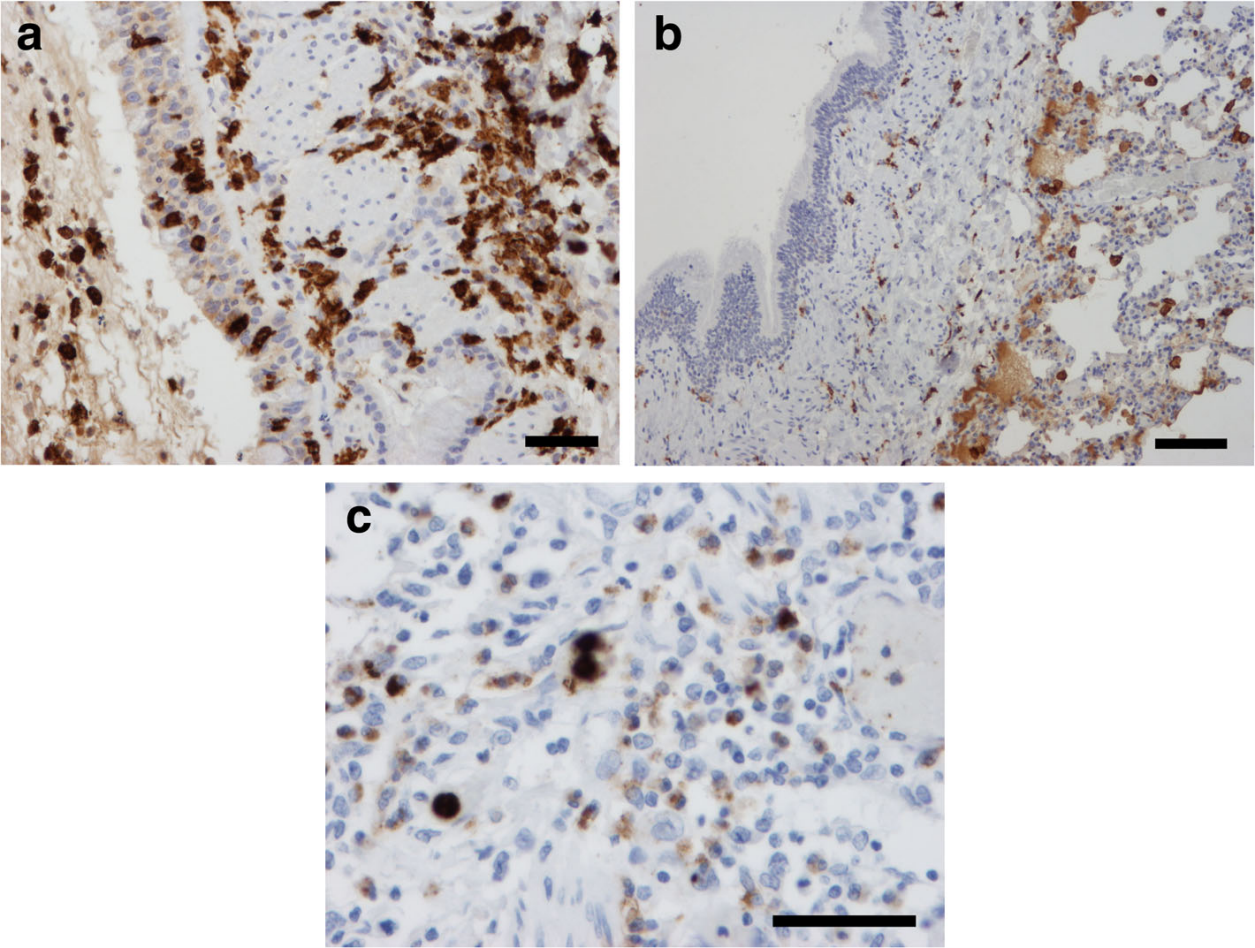
**a** Bronchial macrophages (brown) are increased in epithelium and subepithelium in a 3.6 year old fatal asthma case (CD163; bar 50 um) whereas (**b**) only few bronchial macrophages are found in subepithelium in a 2.5 year old control (bar 100 um). **c** Bronchial mucosa of a 0.9 year old fatal asthma case had numerous eosinophils (lightly positive cells in CD15 staining, brownish) and only a few neutrophils (strongly positive cells in CD15 staining, dark brown) (bar 50 um)

Macrophages (Figs. 5a&b), B-cells, eosinophils and PDCs in large airways were significantly increased in fatal asthma compared to controls, Table 2. In some cases eosinophils were found in large numbers both in airway lumen and mucosa. Eosinophils were easy to identify in hematoxylin-eosin stained sections. Due to degeneration and crushing artefact, neutrophils were difficult to identify and therefore CD15+ cells (including both eosinophils and neutrophils) were counted. In CD15 staining eosinophils stained only lightly in contrast to strongly stainable neutrophils that were counted, Fig. 5c. An effort was made to stain plasma cells with syndecan (CD138) but due to autolysis of autopsy samples even epithelial cells had impaired antigenicity.

Thickness of RBM correlated negatively with numbers of B-cells and mast cells (*r* =−0,692; *p* = 0.023, and *r* =−0.674; *p* = 0.016, Spearman) whereas a significant correlation between numbers of macrophages and B-cells (*r* = 0.790; *p* = 0.002, Spearman) as well between numbers of PDCs and T-cells (*r* = 0.692; *p* = 0.013, Spearman) were seen in fatal asthma. In addition, a significant correlation was detected between numbers of CD15+ cells and macrophages (*r* = 0.648; *p* = 0.023, Spearman) and between CD15+ cells and T-cells (*r* = 0.613; *p* = 0.034, Spearman) in fatal asthma. Numbers of macrophages and B-cells correlated with the length of fatal asthma exacerbation (*r* = 0.664; *p* = 0.026 and *r* = 0.7; *p* = 0.016, Spearman) while number of T-cells correlated with total lifetime duration of asthma symptoms (*p* = 0.636; *r* = 0.035, Spearman).

Mucous plugs were found in large and small airways significantly more prominently in fatal asthma than in controls, especially in large airways (*p* = 0.002, Wilcoxon test), Table 2, Figs. 4c&d.

## Discussion

In this postmortem study, airways of 12 fatal childhood and adolescent asthma cases and 8 controls were evaluated. As anticipated, thickened RBM was found in fatal asthma but contrary to our hypothesis, ASM% was increased only 1/3 of fatal asthma cases, exclusively in large airways. Large airway ASM% increased with age and correlated with RBM and duration of asthma. Our findings also highlight the strong presence of eosinophils in fatal asthma. Moreover, as signs of fulminant inflammation, PDCs, macrophages, B-cells, and amount of mucus were increased in fatal asthma.

### Remodelling

RBM thickness increases during childhood through adolescence in healthy children [3]. The present study confirms these findings adding that the increase is 0.1 um/year. Thickened RBM, the sign of remodeling [4–7], was seen in most of the cases with fatal asthma in this study.

ASM hyperplasia and hypertrophy are thought to discriminate severe asthma from milder disease, and are associated with bronchodilator and increased airway responsiveness [15, 19]. We expected thickened ASM% in both large and small airways in fatal asthma, especially among the oldest patients with longest duration of asthma. ASM% in large airways increased with age only in fatal asthma but there was no difference in median ASM% between asthmatics and controls. Time from death to autopsy and specimen preservation in formalin was more extensive in medico-legal cases used as controls compared to fatal asthma cases. This may have caused autolysis and thereof loosened tissues leading to thicker ASM% in controls.

Since peripheral obstruction is the clinical and functional finding in asthma exacerbation in young children [10] at least some ASM increase in small airways was expected but no increase was detected. The only significant finding in the small airways in fatal asthma cases compared to controls was increased amount of mucus in all but one. Mucus in small airways with luminal diameter < 0.3 mm may contribute to the fatal outcome. To our knowledge there are no reports on ASM in small airways in children with asthma. Recently, small airway ASM was found increased in 41% fatal adult asthmatics whereas pathology limited only to small airways was uncommon [20].

Studies of ASM in severe and fatal childhood asthma are rare. In an observational study, two children with fatal asthma were reported to have thickened RBM and increased bronchial ASM [21]. Similar findings were reported in 4/5 children with non-fatal, difficult-to-control asthma [5]. Bronchial ASM was significantly increased in 24 children (7–16 years) with moderate-to-severe asthma compared to 11 controls (12–49% versus 2–5%) [15]. Both median number size of ASM cells were increased in asthmatics. Our results are partly in accordance with a study of severe therapy-resistant asthma (10–14 years) in which increased bronchial eosinophilia, RBM and ASM mass were found [16]. Increased ASM in severe preschool wheeze was found to discriminate children from those not going to have asthma at school age [22]. The fact that our samples present fairly small airways (median outer luminal diameter in large airways 2 mm and luminal diameter in small airways < 0.3 mm) can also have impact to the low median ASM%.

In the present study the increase of ASM% in large airways with age was greater in fatal asthma cases compared to healthy controls. To our knowledge, there is no published report on this in children. In a study with adults, 18–48 years of age, including patients with fatal and non-fatal asthma and controls, these findings were slightly different [23]. Hypertrophy of ASM cells was found in large airways in both fatal and non-fatal asthmatics whereas hyperplasia of ASM was present in the large and small airways in fatal asthma only. They reported only small or negligible effects of age on ASM cell number or size in fatal asthma.

Here we show that duration of asthma correlated with ASM% in large airways. Similarly, duration of asthma had a small positive effect on ASM area in large airways in adult fatal and non-fatal asthma [23]. It was suggested that increase of ASM occur early in childhood and ASM hyperplasia may contribute to clinical severity. Unfortunately, we did not have the possibility to measure volume or number of ASM cells. Instead we measured ASM area, which is comparative to airway smooth muscle layer thickness, ASM area, used by James et al [23].

### Inflammation

Bronchial eosinophils were not detected in symptomatic children under 2 years of age [10], whereas they were detected in severe wheeze between 2–4 years [24]. In the present study, numerous eosinophils were found in all but one of the fatal asthma cases independently of age. Although findings from adults cannot be translated to children, an increased number of bronchial eosinophils has been a hallmark of severe asthma in adults [20]. In that study, an increased thickness of ASM layer was associated with airway remodelling and eosinophilia but not with neutrophilia [20]. Neutrophils were not increased in our study population either. Mast cells are another prominent cell population in severe adult asthma. Balzar et al. described a predominance of mast cells positive for both tryptase and chymase in the bronchial submucosa and epithelium in adults with severe asthma [25]. In this study, the number of mast cells in large airways was similar in fatal asthma cases and controls.

We found respiratory infection most likely cause of fatal attack. Elevated numbers of bronchial macrophages and B-cells as well association between DCs and T-cells could reflect the acute nature of the fatal exacerbations. We showed also that PDCs were significantly increased in asthmatic airways. Increased numbers of PDCs have been found in human experimental model of allergic rhinitis [26] and in experimental models of asthma in mice [27]. They may play a regulatory role inducing Treg differentiation. PDCs are also involved in defence against various viruses producing IFN-α. However, children with allergic asthma has reduced production of IFN-α by cross-linkage of high affinity IgE-receptor [28]. PDCs are under homeostatic conditions mainly found in secondary lymphoid organs and not in peripheral tissues as lungs. Their accumulation suggests a role in the inflammatory process.

## Conclusions

To our knowledge there are no reports on airway smooth muscle mass in small airways in children with asthma. Undertreated asthma in children and adolescents leads to eosinophilic inflammation, excess of mucus, and remodelling of large airways, i.e. thickened RBM and in minority increased ASM%, but to no other changes in small airways than mucus. Duration of asthma correlated with ASM% in large airways. These findings should alert clinicians to careful examination and proper treatment of children with unstable and difficult asthma.

## Abbreviations

ASM: Airway smooth muscle
PDC: Plasmacytoid dendritic cells
RBM: Reticular basement membrane
